# Metallic Particles in Sodium Battery Anodes: A Review

**DOI:** 10.3390/mi16121391

**Published:** 2025-12-08

**Authors:** Rafaela Ruiz, Carlos Pérez-Vicente, Ricardo Alcántara

**Affiliations:** Department of Inorganic Chemistry and Chemical Engineering, Instituto Químico para la Energía y el Medioambiente (IQUEMA), University of Cordoba, Campus of Rabanales, C3-Building, First Floor, 14071 Córdoba, Spain

**Keywords:** sodium-ion batteries, nanoparticles, energy storage, metals, clusters, electrochemical device, microbatteries

## Abstract

Sodium-ion batteries have emerged as a promising alternative to lithium-ion systems, due to the abundance and low cost of sodium resources. However, the demand for higher performance is always increasing, and developing new electrode materials and optimizing their behavior in full cells is necessary. Their electrochemical performance remains limited by challenges related to the anode materials. A fundamental understanding of electrode materials is essential to advance their practical application, for example, by designing strategies to minimize irreversible processes and enhance the reversible capacity. Thus, the properties of metals, including nanoparticles and clusters, are critical for various types of sodium batteries, such as sodium-ion microbatteries. Additionally, metallic nanoparticles exhibiting special properties are generated in situ at the negative electrode during the electrochemical cycling of certain batteries. This review focuses on their formation mechanisms, structural and electrochemical effects, and strategies to control their distribution and size. Particular attention is given to the interaction between metallic particles and carbon matrices, as well as their influence on capacity. Finally, current limitations and future perspectives for optimizing the properties of the metallic particles in advanced sodium battery anodes are highlighted.

## 1. Introduction

Developing new batteries for renewable energy storage is a serious need. Since sodium is abundant and not costly, SIBs can be handy for a modern and sustainable society [1,2]. On the other hand, since Li can form alloys with Al, but Na does not, SIBs are compatible with low-cost aluminum foils as current collectors, compared to lithium ones that typically employ expensive copper foil at the negative electrode. Although sodium metal has a very high theoretical capacity (1166 mAh g^−1^) and low redox potential (−2.71 V vs. SHE), it is not usually employed in its elemental state as a negative electrode in a battery. The main drawbacks of metallic Na as an anode are an unstable surface, uneven Na deposition, and the risk of dendrite growth, which can lead to capacity fade and the battery’s failure. However, sodium atoms can be chemically combined with metallic and non-metallic elements to provide a more suitable electrode material. The reactions for sodium storage in electrode materials can be classified into three types: intercalation, alloying, and conversion. The last two of those involve metals and/or alloys. Table 1 summarizes the most relevant electrode materials for SIBs, categorized according to their respective reaction mechanisms as discussed in this review. SIBs are most often based on the intercalation of sodium cations into oxides and other compounds. However, metals and alloys are also intimately linked to many of the most promising SIBs. Recent advances in metallic particles for SIBs, along with strategies for their optimization, are summarized in this review. Pure metals, intermetallics, and alloys can be initially employed as electrode materials in SIBs. The sodiophilic metallic elements that have been typically employed in SIBs are in Groups 14, 15, and 16 of the Periodic Table: Sn, Sb, Te, Pb, and Bi. Interestingly, metallic nanoparticles can be in situ formed during the charge/discharge of the battery. For example, controlling the formation of clusters of sodium atoms, with quasimetallic character, in sodiated carbon is highly relevant for developing SIBs.

Since the ionic radius of Na^+^ (102 pm) is relatively large (76 pm for Li^+^), the diffusion of sodium in the solid state is sluggish. Then, compounds in the form of nanoparticles can reduce the path length and improve the kinetics. It is worth noting that the nanosize effect can generate anomalous behavior in properties of metal nanomaterials, such as melting point, crystal structure, and miscibility [43]. On the other hand, the sluggish diffusion kinetics of sodium ions represent a critical challenge for achieving a well-optimized SEI; therefore, rational design and optimization of the SEI are essential. Moreover, the relatively large ionic radius of sodium induces substantial volume variations during the sodiation/desodiation processes, which can lead to severe electrode pulverization and, consequently, rapid battery degradation or failure. To solve this problem, a strategy is to encapsulate the nanoparticles within a stable matrix.

Particularly, the development of sodium-ion microbatteries with 2D or 3D architectures is challenging to achieve [44,45,46,47]. Before the engineering of microelectrodes and microbatteries, it is essential to attain a thorough understanding of the electrochemical mechanisms and associated challenges governing the charge/discharge processes. For example, the repetition of conversion reactions, as well as (de)alloying processes, over many charge/discharge cycles can be detrimental to the structural integrity of electrodes, necessitating the design of strategies to make advanced high-performance microelectrodes.

## 2. Sodium and Interface

Maintaining a stable anode of metallic Na, working properly and safely during many charge/discharge cycles, is challenging [48]. The properties of the SEI built on Na should be electronic insulation, good sodium-ion conductivity, and high mechanical strength. Typically, the main challenges involve the SEI instability and the formation of dendrites, particularly under high current densities. Nevertheless, sodium is less prone to dendrite formation than lithium, and the resulting sodium dendrites are generally softer and pose a lower safety risk. Mechanical stress and strain within the SEI can induce cracking, thereby exposing fresh sodium surfaces to the electrolyte. This exposure accelerates electrolyte decomposition and contributes to the gradual thickening of the SEI layer over repeated cycling. A very thick SEI is impractical for the rapid diffusion of sodium ions. Sodium carbonate and sodium oxide are the main components of the SEI on Na, and NaPF_6_ residue present on the Na surface can form Na_x_PF_y_ and Na_x_PF_y_O_z_ [3]. Using theoretical calculations allows us to select solvents with high LUMO energy levels. For example, FEC with a LUMO value of 1.03 eV is very convenient. Many electrolyte solutions contain F-compounds to induce the formation of a more stable SEI [49,50]. Thus, FEC is a very convenient layer-forming additive. Typically, NaF is formed in the SEI. However, the Na^+^-conductivity of NaF is not very high. Sodium fluoride can create a protective interface on Na and inhibit dendrite formation. For example, Zhou et al. found that nanoparticles of CoF_2_ react in situ with Na, forming NaF and Co on the sodium surface (CoF_2_ + 2 Na → 2NaF + Co), and the NaF/Co hybrid layer is about 15 µm in thickness [4]. The protective layer enhances ion diffusion kinetics and promotes uniform and stable sodium electrodeposition. A minimal NaF content in the SEI and excessive Na_2_O may not effectively protect the Na electrode [3]. It has been suggested that a NaI-rich SEI exhibits a lower energy barrier for sodium diffusion than a NaF-rich SEI, and the use of the CTAI electrolyte has been proposed to achieve such an SEI composition [51]. CTAI helps to create a more stable SEI with a thickness of ca. 40 nm and prevents the formation of dendrites. It was suggested that the upper SEI on Na consists of carbonates, and the inner layer consists mainly of NaF and NaI.

Sodiophilic elements or compounds deposited on metallic Na can create an artificial and more stable SEI. Xie et al. employed silver perchlorate in an organic solvent for creating a layer of Ag crystals on the surface of Na, through the following reaction [5]:

Na + AgClO_4_ → NaClO_4_ + Ag (1)

where the thickness of the artificial layer can be controlled by the soaking time of Na in the silver perchlorate solution.

**Figure 1:**
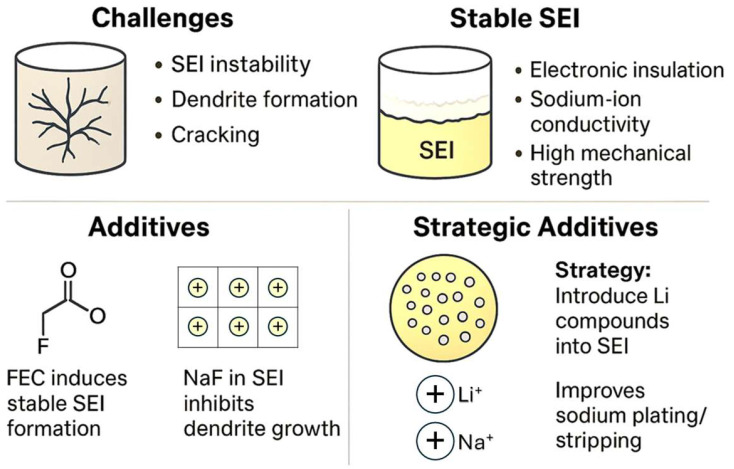
Main issues and strategies of the sodium electrode.

As a novel strategy to stabilize the Na/electrolyte interface and improve the properties of the SEI, it has been proposed to introduce lithium compounds into the SEI of Na by employing certain additives [52]. The SEI contains Li-based compounds (Li_3_N, LiF, and Li_2_CO_3_) formed by the reaction of Na with electrolyte additives such as LiNO_3_ and LiTFSI. With an average thickness of ~887 nm, the SEI remains intact without visible cracking after repeated plating/stripping, while also reducing the polarization during sodium deposition. The challenges and strategies of Na and SEI are summarized in Figure 1.

It has recently been proposed that sodium metal batteries exhibit improved performance over a wide temperature range (−20 °C to 55 °C) when formulated with carefully designed low-concentration electrolytes, for instance, 0.3 M NaPF_6_ in PC, FEC, and TTE [53]. Forming a flexible interphase with a low shear modulus, capable of accommodating the expansion of sodium metal, can contribute to the improvement of sodium plating/stripping. The architecture of gel polymer electrolytes can offer stable interfaces for homogeneous Na plating/stripping [6]. Coating the surface of Na nanoparticles with a layer of tin-sodium alloy can be a sort of interfacial protection engineering to achieve non-dendritic and long-life batteries [54]. Through the careful selection of the solvents in the electrolyte solution, the interface can be modified. For example, a mixture of THF and 2-MeTHF facilitates the cleavage of the P-F bond in P$F_{6}^{-}$ and promotes the formation of a NaF-rich SEI, and improves the cycling stability of microsized Sn electrode [9]. Addition of octamethyltrisiloxane to the electrolyte solution also weakens the P-F bond and facilitates the formation of a NaF-rich SEI [55].

Sodium-potassium alloys exist as a liquid phase over a wide range of compositions (9.2–58.2 wt.% Na at room temperature), and this state can be advantageous for self-healing properties [56]. Anode-less SIBs have also been proposed to reduce the economic cost and to enhance cycling stability. For example, the hard-carbon-derived interphase on an aluminum current collector enables crack-free plating [57].

Bearing in mind that silver is sodiophilic and that TiO_2_ nanotubes exhibit a large surface area, a 3D conductive scaffold of vertically aligned nanotubes of TiO_2_ embedded with Ag nanoparticles can reduce the nucleation barrier for Na plating and prevent dendrite growth, compared to the planar Ti foils [58]. In fact, TiO_2_ nanotubes may be very useful for future microbatteries.

Considering the limitations of experimental studies on clusters of sodium atoms (Na_N_), Huwig et al. computed the most stable isomers for N = 2–150 [59]. These cluster sizes are not sufficiently large to exhibit bulk properties and adopt several atomic arrangements. According to their calculations, the stable structures in the small clusters are not bcc, and a more compact arrangement of atoms (fcc) was suggested. Magic clusters with enhanced stability were identified at N = 74, 83, 87, 95, 104, 131, and 137. Interestingly, through theoretical calculations on the clusters Na_39_, Na_39_^−^, and Na_39_^+^, it was concluded that the electrical charge in the clusters changes their thermodynamic properties, such as specific heat capacities and melting temperatures [60]. It is worth noting that clusters Na_150_ would have a diameter of about 2 nm and can be formed in situ at the discharge of SIBs.

## 3. Sodium Clusters in the Carbon Electrode

Certain sodium properties differ from those of lithium and potassium, for example, the energy of substrate binding, and these different properties affect the ion-intercalation [61]. DFT calculations show that Na–graphite compounds (e.g., NaC_6_, NaC_8_) have positive formation energies and are therefore thermodynamically unstable, unlike their Li or K analogues. The instability arises from the unusually weak substrate/binding energy of Na, which reaches a minimum among alkali metals. This weak binding is a general phenomenon, not specific to graphite, but extended to carbon and other substrates. As a consequence, Na insertion in graphitic materials is intrinsically unfavorable, explaining the low capacity and poor performance of Na-ion batteries with graphite anodes.

On the other hand, hard carbons exhibit different behavior when interacting with alkali metals. According to DFT calculations, the Na-C interaction is substantially weaker than that of the Na-Na interaction, and adsorption of Na on the graphene layer is thermodynamically unstable [62]. In contrast, Li-C and K-C interactions are stronger. This could be due to the relatively higher ionization energy of Na. Consequently, Na has a large tendency to form clusters of atoms with quasimetallic character in the sodiated hard carbon, which is a very common anode material in SIBs. Reduction of sodium ions to form pseudometallic sodium does not occur only at the surface of the electrode exposed to the electrolyte solution, but also within the micropores following the sodium diffusion process [63,64,65], and the pore-filling mechanism is reversible. Thus, the carbonaceous material can act like a sort of matrix that efficiently envelops metallic nanoparticles of Na.

**Figure 2:**
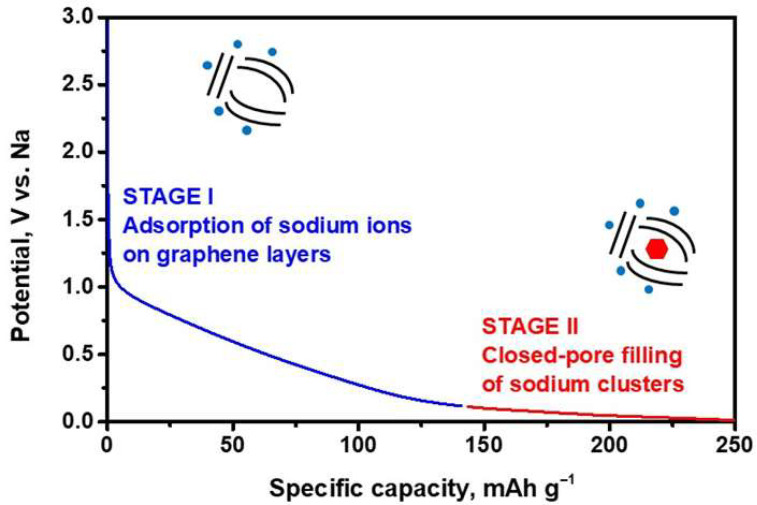
Voltage-capacity curve and schematic diagram of the sodium storage mechanism of a hard-carbon [72,73].

Storage of sodium in carbonaceous materials can proceed through different mechanisms. The sodium initially inserted into the carbon at a high voltage possesses ionic character. Below 0.1 V vs. Na^+^/Na, formation of quasi-metallic clusters of Na has been observed in disordered carbons possessing microporosity [66,67]. For the intercalation of sodium ions between graphene layers in hard carbon, the interlayer spacing must be slightly larger than 3.6 Å [68], whereas “intercalation” for graphene interlayer spacings greater than 4.0 Å should be considered an adsorption process [69]. The presence of quasimetallic Na on the electrodes can be confirmed through a straightforward chemical test: immersing the reduced carbon electrode in ethanol with phenolphthalein results in a red color change and the evolution of H_2_ gas, indicative of reactive sodium species [70]. Pair distribution function analysis of neutron scattering data unveiled a diameter for the sodium clusters of approximately 13–15 Å [71]. Hard carbons with abundant closed pores exhibit a larger capacity for filling these pores with sodium in a quasi-metallic state (Figure 2) [72,73,74,75].

To study the formation of sodium clusters with quasimetallic character in a carbon electrode, several spectroscopic techniques can be employed, for example, EPR [62,75,76,77]. EPR measurements by Kukeva et al. revealed the presence of sodium clusters with diameters between ~2 and 15 nm, whose size distribution depends on the carbon microstructure [78]. The change from ionic sodium to quasimetallic Na near the particle surface can be followed by XPS of the Na 1s core level [79]. The observed shift in the ^23^Na NMR peak during the low-voltage plateau implies that the metallic character of sodium increases [71,80]. In the ^23^Na MAS NMR spectra of sodiated hard carbon, the peak of quasimetallic Na clusters (length > 10 Å) at low voltage is shifted to ca. 660 ppm, while ionic sodium intercalated at higher voltages is detected in the spectra near 0 ppm [71]. Other authors observed the Knight shift of semimetallic sodium clusters at 960 ppm [81,82]. Using in situ XRD, Song et al. detected the reversible formation of Na clusters during filling of closed pores as low-intensity reflections [83]. Pore filling with Na atoms can be followed as an attenuation in the SAXS signal at around 0.01 V, and a reduction in electron density contrast between carbon walls and pore interior [84].

Compared to pure graphite, the interlayer spacing is wider, and the carbon layers are less aligned in hard carbon. A true intercalation of sodium is more favorable, up to the theoretical capacity of 140 mAh g^−1^ (NaC_16_). DFT calculations have found that this intercalation could contribute to the low-voltage plateau (0–0.1 V), although the total capacity of this plateau cannot be explained only by the intercalation [85]. Nanometer-scale Na clusters (3–6 atomic layers thick) lodged between graphene sheets in model micropores are energetically stable, in addition to classical adsorption and intercalation mechanisms. Thus, these nanometer-sized Na clusters stored in carbon micropores can significantly enhance the plateau capacity. The computed formation energies and insertion potentials confirm that such metallic-like Na clustering can account for the unusually high reversible capacity (more than the typical ca. 300 mAh g^−1^ value) observed experimentally (e.g., 478 mAh g^−1^ in MgO-templated hard carbon). Their quasimetallic nature, verified by the Na NMR chemical shifts, facilitates their growth and pore filling, thereby giving rise to the excess capacity already discussed. Meanwhile, the slope capacity (0.1–1.5 V) arises from Na adsorption on defective graphene layers, and the sodium–carbon interaction can be tracked using Raman spectroscopy. The process of sodiation through adsorption on the surface and defective sites can be followed by a decrease in the relative intensity of the Raman band ascribed to disordered regions in carbon, and a red shift in the band ascribed to graphitic regions (transfer of electrons from sodium to graphene sheets) [83,86].

Both the size of the sodium clusters and the careful tuning of the pore size must be considered to optimize sodium storage. It is believed that the smaller the sodium clusters, the higher the carbon capacity. Xie et al. reported that Na clusters forming the plateau close to 0 V are about 1.3–1.5 nm in size, and that smaller pores do not contribute to this low-voltage capacity [86]. Youn et al. found that pores larger than 1.1 nm cannot be completely filled by Na [85]. This voltage region displays diffusion-controlled redox behavior. One key benefit is that closed pores do not increase the carbon–electrolyte contact area, yet their filling with quasi-metallic sodium provides additional capacity near 0 V [87,88,89]. Many micropores that can accommodate Na clusters are closed and not accessible to N_2_ adsorption analysis, making CO_2_ adsorption isotherms necessary for their characterization.

Theoretical simulations of sodium storage in disordered carbon, performed with the ReaxFF force field on a model containing 65,000 carbon atoms, support the notion that the filling of micropores with quasi-metallic sodium occurs during the low-voltage plateau [7]. In this model, the total carbon capacity is 220 mAh g^−1^, and 105 mAh g^−1^ of that capacity corresponds to the filling of the pores with quasi-metallic sodium, which happens after surface adsorption and sodium intercalation. This result highlights the importance of tailoring the pores. A maximum of 6200 atoms of sodium can be accommodated, which is equivalent to Na_0.54_C_6_. Probably, experimental capacities higher than these values would involve the storage of sodium ions through other mechanisms. The simulations also prelude the formation of small Na clusters around vacancies and defect sites.

Xiao et al. studied the relationship between pore size and the voltage at which sodium fills the pores [90]. Closed micropores exhibit a higher potential for Na-filling and reduce the possibility of Na-plating, particularly at high current intensity. The capacity of the plateau near 0 V (formation of quasimetallic Na) does not increase monotonically with the total volume of the closed pores. The ideal size of the closed pores is 1.6 nm. Wang et al., using DFT calculations, elucidated the critical role of pore engineering in sodium storage. The models reveal that a closed pore size of 0.45 nm yields the maximum sodium adsorption energy of −4.87 eV, identifying it as the thermodynamically optimal site for sodium cluster storage. This specific geometry is clearly better than that of both smaller (−0.91 eV) and larger pore configurations (−2.53 eV). On the other hand, optimized architecture features an expanded interlayer spacing of ca. 0.39 nm, which lowers the energy barrier for Na^+^ intercalation and facilitates the ion transport compared to graphite [91].

There are several strategies to create closed pores. Phenolic resins obtained from resorcinol and aldehydes have been employed to create an abundance of closed pores after carbonization and to enhance the storage of quasimetallic sodium [92,93]. To control carbon porosity and enhance the formation of sodium clusters, adding fullerene, C_60_, to the phenolic resin can increase the total volume of the closed pores and generate more sites for sodium storage (plateau capacity) [94]. It seems that C_60_ induces the curvature of graphitic microdomains and thus favors the disordered structure and closed porosity in the hard carbon, while the specific surface area is reduced. Metal oxide particles can act like templates to create pores in the carbons [95,96,97,98,99]. Using in situ template-etching, with zinc gluconate as a templating agent, can generate more closed pores and a large plateau capacity (192 mAh g^−1^) [98]. Zinc gluconate and glucose are carbonized, and nanometric particles of ZnO are formed and then washed off with acid. Zinc gluconate generates closed pores, inhibits the graphitization degree, and expands the interlayer spacing.

It has been discussed that the coexistence of high-potential sloping capacity and low-potential plateau capacity can be detrimental for excellent performance at high rates [100,101], and it has been proposed to integrate materials with sloping voltage curves, such as N/P/S-doped hard carbon embedded with amorphous SnP_x_/SnS_x_ composites [101]. It was observed that it results in an impressive 90% capacity retention over 10,000 cycles. Adding PTCDA to lignin, during the pyrolysis of this mixture to obtain hard carbon, PTCDA molecules connect the lignin-derived carbon skeleton [102]. The resulting hard carbon has an enlarged interlayer spacing (0.397 nm) between graphene layers, and the closed pore structure is reduced. Consequently, sodium storage is dominated by intercalation between the graphene layers, instead of the formation of pseudo-metallic Na clusters. With large interlayer spacing and fewer closed pores, sodium diffusion in hard carbon is more rapid, the contribution of the surface to the capacity is large, and the storage of sodium in the low-voltage plateau is limited. Thus, this type of material is more adequate for a sodium-ion hybrid capacitor [102].

Crystallinity, interlayer spacing, and porosity can be regulated and optimized in carbon materials derived from industrial wastes. Low-temperature (850 °C) pyrolysis and sulfur-doping of carbon derived from petroleum coke, using thiophenic sulfur, enable the development of hierarchical pores [103]. Covalent C-S bonds suppress excessive graphitization, expand the interlayer spacing (0.373 nm), generate defect sites, and facilitate rapid diffusion of sodium towards internal or closed micropores (<2 nm) for storage of quasi-metallic sodium in the voltage plateau. Higher pyrolysis temperatures (e.g., 950 °C) and larger surface area induce carbon framework collapse during cycling, causing pore blockage.

Sequential sulfur-oxygen doping of hard carbon, derived from asphalt, allows a more precise regulation of its microstructure and an extended plateau region [104]. First, sulfur-treatment constructs a 3D disordered network, and then oxygen-treatment promotes denser cross-linking, resulting in the formation of closed-pore microstructures during pyrolysis. SAXS analysis shows that the smallest closed pores have a radius of 1.165 nm, and helium pycnometry indicates a minimum density of 1.68 g cm^−3^. In contrast, pyrolysis without constraints leads to an architecture devoid of closed pores. P_4_O_10_ serves a dual function, acting both as a template for pore-mouth design and closed-nanopore formation, as well as a dopant source [81]. Yang et al. have proposed creating defects (carbon vacancies) in the walls of the closed micropores to increase the plateau capacity and the sloping capacity [8]. To increase carbon vacancies, H_2_ thermal reduction was employed. The reversible capacity is 470 mAh g^−1^, including a plateau region with a capacity of 299 mAh g^−1^.

These results demonstrate that the carbon-based matrix plays a crucial role in controlling the nucleation and growth of sodium nanoparticles and clusters within the anode of sodium-ion batteries (SIBs). Nevertheless, a serious safety issue has been recently published. Using NMR, Cui et al. found that the sodium clusters exhibit significant metallic character, with even more conduction electrons at the Fermi energy level than bulk Na, making them highly reactive and accelerating thermal runaway [105].

## 4. Sodium-Alloying

### 4.1. Tin

**Figure 3:**
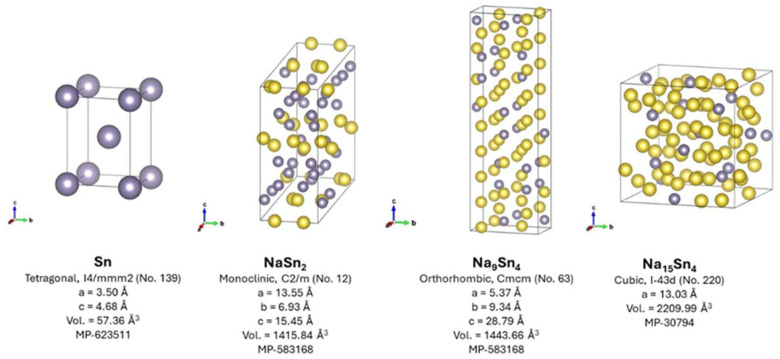
Crystal structures for tin and sodium-tin intermetallics obtained from the Materials Project database [106].

Silicon and germanium have relatively low ability to form alloys with sodium and/or very sluggish kinetics for sodium diffusion, and consequently, tin is the lightest element in group 14 with a significant theoretical capacity in a sodium cell (847 mAh g^−1^). To illustrate their different dimensions, the crystal structures of the main intermetallic sodium-tin compounds are shown in Figure 3. The total electrochemical reaction can be written as:

4Sn + 15Na → Na_15_Sn_4_ (2)

The substantial volume expansion (about 420% for Na_15_Sn_4_ relative to Sn) that occurs during charge/discharge cycling induces severe mechanical stress, resulting in electrode pulverization and eventual battery failure [107]. One strategy to mitigate the volume changes occurring during the formation of tin-sodium alloys is to mix, or alloy, tin with other elements such as cobalt or carbon. Another approach involves reducing the initial particle size (typically to around 10 nm); however, smaller particles exhibit a larger surface area in contact with the electrolyte, which promotes irreversible electrolyte decomposition during SEI formation and consequently reduces the coulombic efficiency. Interestingly, recent studies have shown that, in ether-based electrolytes, micrometric Sn exhibits better cycling stability than its nanometric counterpart [10]. A high Sn-charge (80%) in tin-carbon composites is better when diglyme-based electrolytes (676 mAh g^−1^ after 70 cycles at 0.1 A g^−1^), and the reason for that can be the formation of an extended coral-like framework by Sn that enables connectivity during the cycling.

Within the strategies for high-performance tin-based electrodes, metallic substrates with non-planar geometry can help to achieve electrodes with higher areal capacities. Thus, Co-Sn alloy can be electrodeposited on nickel-foam [108]. Ultrafast heating of fructose and SnO_2_ can form nanoparticles of Sn surrounded by a carbon matrix with good electrochemical cycling [109]. Rapid heating is critical for obtaining small Sn particles and good electrochemical properties. A hierarchical yolk-shell structure composed of Sn-yolk and carbon/silicon oxycarbide bilayer shell stabilizes the electrode/electrolyte interface [110]. Void interspaces inside the yolk-shell nanohybrid accommodate the volume change of the Sn yolk during cycling. Another interesting microstructure is tin nanospheres anchored on and embedded in carbon nanofibers [111]. Alloys may also be incorporated as electrode additives to enhance electrochemical performance. To increase the initial coulombic efficiency of the SIB, the intermetallic compound Na_15_Sn_4_, mixed with hard carbon, has been proposed as a sodium reservoir to compensate for the irreversible sodiation in the first cycle of the carbon [112].

Examination of the electrode particles after electrochemical cycling can unveil surprising behaviors. It has been found that optimizing cell configuration, for example, by using a double nanoporous separator as a physical barrier, Sn powder exhibits a self-healing phenomenon during cycling [113]. Although the Sn powder undergoes pulverization during the initial (de)sodiation cycle, the in situ formed Sn particles with a porous coral-like morphology exhibit mechanical stability against volume fluctuations and preserve electrical contact. The MWCNT conductive additive establishes efficient conductive pathways between Sn particles, outperforming Super P carbon.

### 4.2. Antimony

The formation of Na_3_Sb delivers a high theoretical capacity of 660 mAh g^−1^, presenting significant potential for sodium-ion battery applications. Nevertheless, the substantial volume expansion of about 390% compared to pure Sb remains a critical limitation, motivating the development of advanced synthesis approaches to produce micro- or nanoscale particles. In contrast to Sb nanoparticles, atomically dispersed Sb species act as superior nucleation centers for the reversible growth of Na–Sb alloy phases such as Na_3_Sb [11]. Via electrospinning, the metallic Sb core was encapsulated by the MoS_2_ shell (Sb@MoS_2_), and the Sb@MoS_2_ nanoparticles were embedded in carbon nanofibers [12]. Dispersing nanoparticles of a compound with Na-alloying elements (SnSb of about 10 nm) in a carbon matrix can provide an electrode with a very high capacity (544 mAh g^−1^) [13]. Nanoparticles of Sn-Ge-Sb alloy (10–15 nm) can also deliver exceptionally high capacity and cycling stability (662 mAh g^−1^ after 50 cycles) [14]. The hollow structure of carbon nanotubes can encapsulate nanoparticles of Sb [114] and Sb_2_O_3_ and accommodate the large volume changes [115]. Electrospinning was employed to fabricate a mosaic distribution of Sb/P nanospheres, with sizes ranging from 100 to 200 nm, embedded within a carbon fiber matrix. During sodiation, the formation of amorphous Na_3_Sb and Na_3_P was assumed to occur [116].

The theoretical capacity of Sb_2_O_3_ in a conversion reaction is as high as 1109 mAh g^−1^. Zheng et al. confirmed the transformation of Sb_2_O_3_ into Na_2_O and Na_3_Sb by TEM, XRD, and XPS [115]. During desodiation, Na_3_Sb is converted back into crystalline Sb and Sb_2_O_3_. Regulating the crystallographic facets exposed to the electrolyte solution can be employed to enhance the electrochemical performance. Zheng et al. prepared nanobelts of Sb_2_O_3_ with exposed (010) facets [117]. The polymer PVP promotes the anisotropic growth of Sb_2_O_3_ particles with a nanobelt morphology and the exposure of the (010) crystallographic plane, which has a large spacing. This nanoarchitecture decreases the Na-driven volume expansion and improves sodium storage capability. It was proposed that starting from a mixture of Na_3_Sb and Na_2_O, during the charge process, firstly Na_3_Sb dealloys to Sb, and then further charging and desodiation transform the mixture Sb-Na_2_O into Sb_2_O_3_.

### 4.3. Tellurium

Tellurium has an excellent electronic conductivity (2 × 10^2^ S cm^−1^), high density (6.2 g cm^−3^), and a high theoretical specific capacity (420 mAh g^−1^), resulting in an outstanding theoretical volumetric capacity for SIB (2621 mAh cm^−3^) [15,16]. These intrinsic properties make Te-based materials highly attractive as high-energy-density anodes. However, the practical application of pure Te is hindered by several critical challenges. Like other chalcogen-based materials, elemental tellurium is prone to polychalcogenide shuttle behavior during cycling, which leads to active material loss and poor Coulombic efficiency. In addition, Te undergoes substantial volume expansion upon sodiation, which can cause particle pulverization, loss of electrical contact, and rapid capacity decay. To overcome these limitations, researchers have increasingly focused on metal tellurides and carbon–telluride composites, which offer improved structural stability and optimized reaction kinetics. Metal tellurides, such as FeTe_2_, NiTe_2_, or CoTe_2_, typically exhibit stronger metal–Te bonding and more favorable conversion or alloying mechanisms, leading to reduced shuttle effects and enhanced cycling durability. Likewise, integrating Te or metal tellurides within conductive carbon frameworks—such as carbon nanofibers, graphene, or porous carbons—provides mechanical buffering, improved electron/ion transport pathways, and confinement of telluride species. For example, FeTe_2_ nanoparticles embedded in carbon nanofibers, and prepared using the electrospinning technique, have demonstrated remarkable electrochemical resilience, delivering stable performance over 1000 cycles [17]. This behavior is attributed to the synergistic combination of the robust Fe-Te structure and the flexible, conductive carbon matrix, which effectively buffers volume changes and preserves electrode integrity during repeated sodiation/desodiation processes.

### 4.4. Bismuth

The maximum theoretical capacity of bismuth is relatively modest (385 mAh g^−1^ for Na_3_Bi) due to its high atomic mass. In recent years, the economic cost of Bi has risen significantly, making it less competitive compared to tin. Nevertheless, several research groups have continued to investigate its potential. In the Bi-Na system, intermediate compositions (tetragonal NaBi) and several structures (cubic and hexagonal Na_3_Bi) can be formed [18]. The sodiation process and structure evolution involve dramatic volume changes. Nanoparticles of Bi reduce the diffusion path length of sodium but tend to agglomerate during electrochemical cycling.

Like other Na-alloying elements, encapsulation of Bi nanoparticles in carbon matrices is being explored. For example, carbon fibers [19]. Hollow carbon spheres are highly effective, even over 20,000 cycles [20], and Bi nanoparticles were observed inside the hollow carbon spheres after electrochemical cycling. Very often, the behavior of SIBs at very low temperatures is very poor. However, Bai et al. reported that Bi nanoparticles embedded in carbon nanorods have a capacity of 238 mAh g^−1^ at 2 A g^−1^ and −40 °C [21]. Since Sb is lighter than Bi, the Sb-Bi alloys can have a higher gravimetric capacity than pure Bi. Although Sb can be dissolved and shuttled, Bi crystals tend to fix Sb atoms. Porous carbon can attenuate the issues of the Sb-Bi alloy [22]. The ultrafast high-temperature shock method has been employed to prepare Bi nanoparticles (average particle size ca. 26 nm) embedded in carbon nanorods, [21]. The activation of the Bi electrode through high-rate cycling at low temperature was found to increase the specific surface area, generate new electrochemically active sites, and consequently improve the capacity. As an alternative to encapsulating Bi nanoparticles, it has been proposed to create Bi nanotubes. For this purpose, Pu et al. employed iodine-assisted galvanic replacement, BiI_3_ as a bismuth precursor, and copper nanowires as templates and reducing agents [23]. The formation of the Bi nanotubes was based on the Kirkendall effect. The cycling stability was impressive: 355 mAh g^−1^ after 15,000 cycles at 20 A g^−1^. Bi nanoparticles (35–75 nm) dispersed in a silica matrix are formed by heat-treatment of CuO-Bi_2_O_3_-SiO_2_ glass in a reducing atmosphere of H_2_, and this material serves as an electrode for sodium batteries [24]. The silica matrix inhibits the dissolution of metallic particles.

Bismuth has a layer structure and can be exfoliated into 2D nanosheets, forming a few-layer structure and even a monolayer structure. Analogous to graphene, the monolayer of bismuth is named bismuthene, with a theoretical thickness of 0.395 nm [118]. Bismuthene/graphene heterostructure can exhibit improved properties [119]. According to theoretical calculations, increasing the concentration of Na in bismuthene involves clustering Na atoms together, and it becomes energetically more favorable than binding to bismuthene, which increases the risk of dendritic growth. However, the heterostructure graphene/bismuthene inhibits the growth of dendrites and improves the electronic conductivity.

Bismuth oxide can work like a conversion-type material, and the Na-alloying capacity of the in situ-formed Bi metal particles can increase the overall capacity (theoretically up to 670 mAh g^−1^). Mu et al. have proposed to use a composite electrode material containing Bi_2_O_3_ and reduced graphene oxide [120]. The flexibility of the graphene structure accommodates the volume changes. The Bi/Bi_2_O_3_ heterojunction spontaneously generates internal electric fields that facilitate the transfer of electrons and ions.

### 4.5. Binders for Metals

Binders play a critical role in enhancing the electrochemical performance and mechanical integrity of sodium-ion batteries. In particular, they help mitigate the substantial volume changes associated with alloy-type anode materials, thereby improving electrode stability, adhesion, and cycling durability. It has been reported that the most popular polymer binder for LIBs, PVDF, is not very suitable for the anodes of SIBs, particularly Na-alloying anodes such as Sn, Sb, and Bi; therefore, alternative binders should be used [121,122]. For example, cross-linking of PAA with GLY has been proposed. The tensile resistance of PAA-GLY surpasses that of PAA and PVDF, and tin electrodes using PAA-GLY as a binder exhibit enhanced stability. The cross-linking of PAA-GLY increases the Young’s modulus of the electrode. Notably, this binder enables the efficient utilization of Bi microparticles even in the absence of dispersion within the carbon material.

## 5. Formation of Metallic Particles Through Conversion-Type Electrodes

Conversion-type electrodes, including metal oxides and other reducible compounds, undergo reduction by sodium to yield metallic phases and sodium compounds. The sodium stored within the electrode can subsequently be released through oxidation. While the multivalence of the metal contributes to high theoretical capacities, the pronounced lattice-volume variation during redox cycling often leads to structural degradation. Gaining insight into these reaction mechanisms is crucial for designing strategies to improve long-term cycling stability.

### 5.1. Transition-Metal Oxides

**Figure 4:**
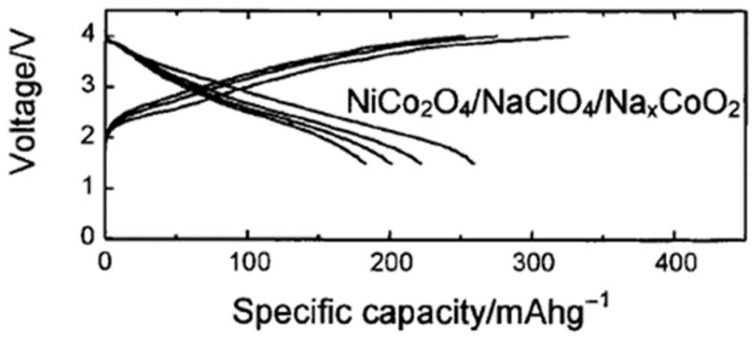
Galvanostatic charge/discharge cycles for a full SIB NiCo_2_O_4_/Na_x_CoO_2_ [106]. Reproduced under ACS License No. 6147051118158.

Bimetallic oxides are particularly promising, as their capacities can exceed those of hard carbon, making them suitable for use in SIBs (Figure 4). For the electrochemical reaction between NiCo_2_O_4_ and sodium down to ca. 0.0 V vs. Na^+^/Na, a mechanism mainly based on conversion reactions was first proposed in the year 2002 and later studied in more depth [25,26,123,124,125,126,127,128,129,130,131,132]:

NiCo_2_O_4_ + 8Na → Ni + 2Co + 4Na_2_O (3)

Ni + 2Co + 4Na_2_O ⇌ NiO_x_ + 2CoO_y_ + (2x + 4y)Na + (4 − x −2y)Na_2_O (4)

where reaction (3) is irreversible, and NiCo_2_O_4_ is not recuperated, while reaction (4) is reversible with x ≅ 1.0 and y ≅ 1.0. On the other hand, reaction (3) could be divided into three sodiation steps:

– Step 1. Veritable insertion of sodium ions into the host structure of the spinel in the range between ca. 3.0 and ca. 0.8 V. This step remains little explored.
– Step 2. Destruction of the spinel structure and formation of NiO, CoO, and Na_2_O, at around 0.8–0.2 V. This intermediate step was particularly corroborated by Zhu et al. [127], using in situ TEM and electron diffraction patterns.
– Step 3. Reduction of the oxides into metallic nickel and metallic cobalt, and further formation of sodium oxide near 0.0 V. This reduction process has been reported by several authors [25,123,124,125]. Thus, Zhu et al. observed the diffraction rings of metallic Co, Ni, and Na_2_O in the electron diffraction images of the fully sodiated product [123].

The primary challenge of this electrode material is that the substantial volume changes of the crystal lattice can adversely affect electrochemical cycling. Since the ionic radius of sodium is larger than that of lithium, this limitation is even more pronounced in SIBs. A typical strategy to overcome this drawback is to employ nanoparticles and special nanostructures that could buffer the volume changes and alleviate the strain during the charge/discharge cycle [26,128,129,130,131]. Lee et al. grew NiCo_2_O_4_ directly on Ni foam and studied the conversion mechanism in both lithium and sodium cells [132]. After the formation of Ni and Co, it was found that Co_3_O_4_ was reformed during the oxidation in the Li cell, but CoO was formed in the Na cell.

After NiCo_2_O_4_, many other transition-metal oxides have been reported in the literature as conversion-type anodes for sodium-ion batteries. For example, in a paper by López et al. Na_3_V_2_(PO_4_)_3_ was employed as a positive electrode, and the negative electrode was a thin film of NiO and Fe_2_O_3_ [133]. It has been assumed that during the reaction of CoFe_2_O_4_ in a sodium cell, Fe and Co are first in situ formed and then Fe^3+^ and Co^2+^ are regenerated (in the form of CoO and Fe_2_O_3_) during the desodiation process, although the direct evidence of that still has not been checked [27,28,29]. Strong surface capacitance was detected by magnetometry in the reduced metallic nanoparticles, and this charge on the particle surface can be a source of extra capacity in the battery [134,135]. This effect can justify that the decrease in particle size increases the initial capacity. The formation of metallic Ni and Na_2_O after the conversion reaction of NiO was corroborated by using ex situ XRD, XPS, and magnetization measurements [30].

The morphology of the particles strongly influences the reaction mechanism with Na and the formation of metallic particles. Thus, Vincent et al. demonstrated that Co_3_O_4_, composed of nanoparticles with a spherical morphology, exhibits capacitive-type charge storage rather than a conversion-based mechanism [31]. These particles could facilitate surface charge storage, which is not limited by ion diffusion. This contrasts with diffusion-controlled conversion, for example, in Co_3_O_4_ with nanosheet morphology, which undergoes a conversion reaction. Defective oxides can exhibit modified properties. The spinel-type Fe_3_O_4_ was prepared with cation vacancies through Mo^4+^-doping [136]. Iron vacancies are created to balance the electrical charge. This defective anode Fe_3−4x/3□x/3_Mo_x_O_4_ (x ≤ 0.20) exhibits improved capacity retention and rate capability compared to defect-free Fe_3_O_4_. The cation vacancies enhance sodium intercalation, and the conversion process is partial even after 150 cycles. The theoretical capacity of CoNiO_2_ is 716 mAh g^−1^, and in the form of 3D rose-like morphology, it possesses an outstanding electrochemical reaction kinetics (343.5 mAh g^−1^ following 100 cycles at 0.5 A g^−1^) [32].

### 5.2. Transition-Metal Phosphides

Transition metal phosphides (FeP, Ni_2_P, Cu_3_P…) react with sodium and sodium phosphides are formed (Ni_2_P + 3Na = Na_3_P + 2Ni). NaP is produced by the transformation of Na_3_P (Na_3_P = 2Na + NaP). Composite electrodes containing several phosphides have been studied [137]. Special morphologies and a carbon matrix can also contribute to buffer volume changes during discharge/charge of the phosphides. In the sodiated FeP@carbon nanocomposite, the formation of metallic Fe and Na_3_P phases was corroborated by SAED [33]. Their capacity is limited, while phosphides of Na-alloying elements (e.g., Sn) can deliver higher capacities than transition-metal phosphides [34] and can be classified into another category.

### 5.3. Metal Sulfides

Ferric pyrite could be a cheap and non-toxic substance for a conversion-type anode with high capacity, following these reactions:

FeS_2_ + xNa → Na_x_FeS_2_ (5)

NaFeS_2_ + Na → Na_2_S + Fe (6)

with the intermediate formation of Na_x_FeS_2_ intercalation compound. During the charge process, the discharge products, Na_2_S and Fe, are not reconverted into FeS_2_. Soluble polysulfides (Na_2_S_x_) are formed, and these lead to the polysulfide effect and electrode failure [138]. Then, strategies have been adopted to suppress the polysulfide effect. Nitrogen-doped carbon nanosheet has been employed as an optimized substrate to support FeS_2_ nanoparticles, and a remarkable cycling stability (2200 cycles) was achieved [139]. A metal–organic framework was employed as a precursor and template to prepare pyrrhotite (Fe_1−x_S) encapsulated by N, S-co-doped carbon [140]. The carbon matrix functions as an adsorbent for polysulfides, thereby reducing the shuttling effect, and also acts as a physical shield that protects the copper foil (current collector) from corrosion. The aluminum foil current collector also experiences corrosion because of the shuttle effect [138]. A Mott-Schottky heterojunction formed between FeS_2_ and N-doped carbon nanofibers could enable fast electronic/ionic transfer [35]. The junction establishes a built-in electric field that drives charge redistribution at the interface, promoting efficient charge transfer during (de)sodiation. After the intercalation of sodium into MoS_2_, the conversion reaction and formation of Na_2_S and metallic Mo have been proposed [36,37,38,39]. The method of synthesis and, particularly, the source of sulfur, influence the properties of the cobalt sulfides CoS_x_, and it seems that the compound CoS_2_ has better electrochemical performance and more efficient conversion-reaction [40]. Cobalt sulfide nanoparticles embedded in carbon layers, forming a core–shell structure (Co_9_S_8_@Co/C), can be reduced by sodium reversibly forming metallic Co and Na_2_S [41]. During charging, the reaction rate of metallic Co with Na_2_S is slower than the conversion of sodium sulfide into polysulfides, resulting in the shuttle effect (migration of polysulfides within the electrolyte). Fortunately, the carbon shell mitigates sulfide and polysulfide dissolution in the electrolyte, thereby suppressing the shuttle effect.

Sulfides of Na-alloying metals, such as Sn and Zn, could have larger capacities. In fact, monolayer tin disulfide SnS_2_ is an intrinsically appealing anode material for rechargeable sodium-ion batteries due to its high theoretical capacity and layered structure. However, its practical application is fundamentally hindered by two major drawbacks: severe volumetric expansion during sodiation and a wide electronic band gap that limits charge transfer kinetics. These drawbacks can be overcome with SnS_2_/graphene heterostructures [141,142,143]. DFT calculations were used to model the sodiation thermodynamics and kinetics in a monolayer of SnS_2_ and in a SnS_2_/graphene heterostructure. The pristine monolayer exhibited a high theoretical capacity of 586 mA h g^−1^ and a remarkably low Na^+^ diffusion barrier of 0.13 eV, but it was constrained by a ca. 20% area expansion and a large electronic band gap (1.54 eV). The formation of the SnS_2_/graphene heterostructure increased the binding energy of Na at the interface, simultaneously resulting in a metallic character and effectively decreasing the volume expansion to 2.4%. Although the interface diffusion barrier slightly increased to 0.26 eV, the resulting composite is computationally predicted to be a high-performance, mechanically stable, and conductive anode material for SIBs [142].

Another closely related approach is to encapsulate nanoparticles of SnS_2_ by carbon nanofibers. These materials can maintain a capacity of ca. 650 mAh g^−1^ [141]. DFT calculations were again performed to elucidate the synergistic role of the SnS_2_/C heterostructure in sodium storage. Interfacial charge density difference maps confirmed significant electronic coupling, predicting enhanced electronic conductivity due to favorable charge redistribution between the SnS_2_ nanoparticles and the conductive carbon matrix. DFT calculations further revealed a low energy barrier for Na^+^ diffusion (0.25 eV, a similar value to that computed when using graphene [142]) within the SnS_2_ lattice, confirming the rapid Na^+^ ion kinetics in this material. The strong SnS_2_/C interfacial binding energy theoretically stabilizes the conversion/alloying intermediates and suggests that the carbon encapsulation effectively mitigates the strong volume expansion stress inherent to the sodiation process [142].

The formation of a Zn, Na_2_S, and Na_x_Zn alloy, through the conversion of ZnS, has been confirmed [144]:

ZnS + 2 Na → Na_2_S + Zn (0.5 V) (7)

Zn + xNa → Na_x_Zn (0.5–0.01 V) (8)

When certain types of biomass are used as a carbon precursor to form a carbon-ZnS composite, a nitrogen-doped carbon can be formed. The highly electronegative nitrogen atoms can irreversibly trap sodium atoms, thereby decreasing the columbic efficiency [144].

Another strategy is the use of a high-entropy conversion-type electrode, such as the sulfoselenide Cu_0.88_Sn_0.02_Sb_0.02_Bi_0.02_Mn_0.02_S_0.9_Se_0.1_, with a reversible capacity of 325 mAh g^−1^ after 10,000 cycles at 30 A g^−1^ [42]. The resulting particle morphology suggests that the high-entropy configuration reduces the energy for surface formation. Many metal elements can undergo sequential electrochemical reactions and prevent the instantaneous collapse of the structure. The experimental results confirmed the conversion reaction and formation of Cu, Mn, Na_2_S, Na_2_Se, Na_9_Sn_4_, Na_3_Sb, and Na_3_Bi.

## 6. Innovative Directions

A conversion-reaction has been employed in a light-driven rechargeable battery, combining energy harvesting with energy storage in a cell made of an exfoliated MoSe_2_ electrode and a Na metal electrode [145]. The discharge/charge reactions are given below.

First reduction:

xNa + MoSe_2_ → Na_x_MoSe_2_ (intercalation at ca. 0.6 V vs. Na) (9)

Na_x_MoSe_2_ → Na2Se + Mo (conversion at ca. 0.3 V vs. Na) (10)

Oxidation:

2Na_2_Se + Mo → MoSe_2_ + 4Na (11)

Second reduction: from Se to Na_2_Se_n_ (4 < n < 8) and then to Na_2_Se_2_. Second oxidation: from Na_2_Se to Se.

The peak current in the cyclic voltammograms increases under illumination. The light generates charge carriers that assist the charge reaction. Electron holes are photogenerated in the valence band, and these holes repulse the sodium intercalated into the electrolyte, desodiating and generating MoSe_2_.

As an alternative to conventional alloys, high-entropy alloys can be employed. This type of alloy contains at least five elements randomly distributed in the same crystal structure, to provide high capacity for Na-alloying and to exhibit improved mechanical properties. A high-entropy Bi_x_Sn_y_Sb_y_Cu_x_Al_x_ nanoalloy embedded in porous carbon fibers, and fabricated by the electrospinning method, was reported by Liu et al. [146]. The metallic nanoparticles, with diameters around 50–100 nm, were dispersed in carbon fibers with diameters of 200–300 nm. The concept of high entropy could be applied to other types of battery materials, not only alloys, for example, oxides.

It is worth noting that hard carbon can even be suitable as an anode for all-stretchable SIB [147]. However, Na-alloys would require sophisticated microengineering and innovative three-dimensional microarchitecture.

A 3D carbon architecture can mitigate the volume changes of the Na-alloying microelectrode, and the semiconductor industry’s technology can be applied to the fabrication of microelectrodes. Interestingly, Yue et al. first fabricated a square Si micro-rods array using photolithographic technique and etching, then employed plasma-enhanced chemical vapor deposition (PECVD) to grow graphene on the Si rods, and finally, an Sb shell was deposited by radio frequency magnetron sputtering (RF-MS) [148]. The resulting 3D silicon/graphene/antimony microrod array has excellent cycle stability in SIB. Additionally, 3D printing fabrication could be more convenient for future SIB microbatteries, although this area remains scarcely explored, and the observed capacities are moderate [149,150,151].

## 7. Conclusions

**Figure 5:**
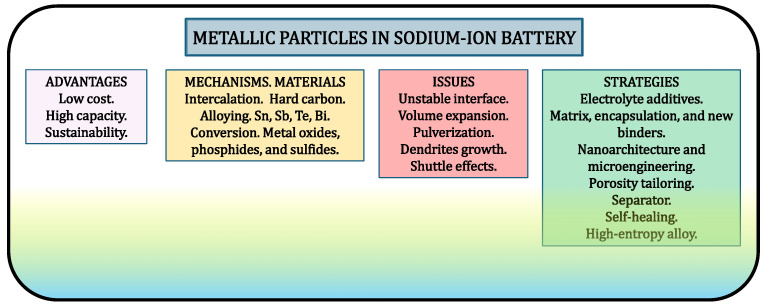
Schematic overview of the key aspects of metallic particles in SIBs.

A schematic illustration of the principal aspects discussed above is shown in Figure 5. This study highlights that metals offer several advantages as components in sodium-ion batteries (SIBs). The conventional copper current collector can be replaced with more corrosion-resistant metals such as aluminum or titanium, while tuning particle size and morphology enables the tailoring of electrochemical behavior and the controlled generation of metallic species within electrodes. The main reaction mechanisms that lead to the in situ formation of metallic particles include (i) pore filling by quasi-metallic sodium in hard carbon through an intercalation reaction, (ii) conversion reactions of metal oxides, and (iii) alloying with other metals such as tin. At present, the use of hard carbon as an anode remains the most realistic, economical, and straightforward approach. However, recent progress regarding the other two mechanisms suggests that they could become viable alternatives if the synthesis routes for these emerging materials become more accessible. Moreover, metallic nanoparticles formed during conversion reactions can contribute additional capacity through surface charge accumulation, and the design of hard carbons with closed pores provides a means to regulate the formation of quasimetallic sodium clusters. Much effort is devoted to designing carbon materials with optimized pores capable of storing sodium clusters. The use of cross-linked polymer binders also represents a promising strategy to mitigate the mechanical degradation of Na-alloying particles. DFT calculations are essential for guiding the design of high-performance SIB materials, from electrolyte additives and SEI chemistry to hard-carbon anodes and metal-sulfide nanostructures. By revealing sodium’s unique interactions at the atomic scale, DFT enables the prediction of optimal pore sizes, interlayer spacings, and composite architectures. The theoretical insights not only clarify the mechanisms of Na storage but also accelerate the development of safer, more efficient SIB technologies and advanced metal-based nanomaterials. Nevertheless, significant challenges remain, including the large irreversible capacity loss during the first cycle, the pronounced volume changes associated with conversion reactions, and the heavy nature of Na-alloying elements. Additional concerns involve parasitic reactions such as shuttle effects in metal sulfide electrodes, the limited suitability of common LIB binders for metallic SIB electrodes, and potential safety risks related to the instability of quasimetallic sodium clusters that may trigger thermal runaway. Future research should focus on exploring the in situ formation and evolution of metallic nanoparticles during cycling, controlling exposed crystallographic facets to enhance electrochemical performance, and developing composite electrodes that exploit synergistic effects between different materials. One critical factor for achieving remarkable progress is the design of binders that can accommodate reversible metallic particle formation while buffering volume variations. Promising directions also include the design of self-healing electrodes, the application of high-entropy alloys, and the use of advanced 3D-printing techniques to create novel architectures for high-performance sodium-ion microbatteries.

## Figures and Tables

**Table 1:** Summary of the most significant anode materials for SIBs involving metallic nanoparticles.

Type	Compound	Calculated Capacity, mAh g^−1^	Experimental Capacity, mAh g^−1^	References
Metal	Na	1166	-	[3,4,5,6]
Intercalation	hard carbon	140 (NaC_16_) + 105 (pores)	172 (sloping) + 299 (plateau)	[7,8]
Alloy	Sn	847	676 (Sn-C)	[9,10]
	Sb	660	662	[11,12,13,14]
	Te	420	338 (Te-C)	[15,16,17]
	Bi	385	355	[18,19,20,21,22,23,24]
Conversion	NiCo_2_O_4_	890	701	[25,26]
	CoFe_2_O_4_	910	390	[27,28,29]
	NiO	720	250	[30]
	Co_3_O_4_	890	622	[31]
	CoNiO_2_	716	385	[32]
	FeP	926	656 (FeP-C)	[33,34]
	FeS_2_	894	629	[35]
	MoS_2_	335	338 (MoS_2_-C)	[36,37,38,39]
	CoS_2_	870	833	[40,41]
	Cu_0.88_Sn_0.02_Sb_0.02_Bi_0.02_Mn_0.02_S_0.9_Se_0.1_	-	583	[42]

## Data Availability

No new data were created or analyzed in this study. Data sharing is not applicable to this article.

## Abbreviations

The following abbreviations are used in this manuscript:

SIBs	Sodium-ion batteries
SHE	Standard hydrogen electrode
LIBs	Lithium-ion batteries
SEI	Solid electrolyte interface
FEC	Fluoroethylene carbonate
LUMO	Lowest unoccupied molecular orbital
CTAI	Cetyltrimethylammonium iodide
LiTFSI	Lithium bis(trifluoromethanesulfonyl)imide
PC	Propylene carbonate
TTE	1,1,2,2-tetrafluoroethyl 2,2,3,3-tetrafluoropropyl ether
THF	Tetrahydrofuran
2-MeTHF	2-methyltetrahydrofuran
DFT	Density Functional Theory
EPR	Electron Paramagnetic Resonance
XPS	X-ray Photoelectron Spectroscopy
NMR	Nuclear Magnetic Resonance
MASNMR	Magic Angle Spinning Nuclear Magnetic Resonance
XRD	X-Ray Diffraction
SAXS	Small-Angle X-Ray Scattering
ReaxFF	Reactive Force Field
PTCDA	Perylene-3,4,9,10-tetracarboxylic dianhydride
PAA	Poly (acrylic acid)
GLY	Glycerin
PVP	Polyvinylpyrrolidone
PVDF	Poly (vinylidene fluoride)
MWCNT	Multi-walled carbon nanotubes
TEM	Transmission Electron Microscopy
SAED	Selected area electron diffraction
PECVD	Plasma-enhanced Chemical Vapor Deposition
